# Auto-Spikformer: Spikformer architecture search

**DOI:** 10.3389/fnins.2024.1372257

**Published:** 2024-07-23

**Authors:** Kaiwei Che, Zhaokun Zhou, Jun Niu, Zhengyu Ma, Wei Fang, Yanqi Chen, Shuaijie Shen, Li Yuan, Yonghong Tian

**Affiliations:** ^1^School of Electronic and Computer Engineering, Shenzhen Graduate School, Peking University, Shenzhen, Guangdong, China; ^2^Peng Cheng Laboratory, Shenzhen, Guangdong, China; ^3^Department of Computer Science and Engineering, Southern University of Science and Technology, Shenzhen, Guangdong, China

**Keywords:** spiking neural network (SNN), transformer, transformer architecture search, network architecture search (NAS), evolutionary algorithm (EA)

## Abstract

**Introduction:**

The integration of self-attention mechanisms into Spiking Neural Networks (SNNs) has garnered considerable interest in the realm of advanced deep learning, primarily due to their biological properties. Recent advancements in SNN architecture, such as Spikformer, have demonstrated promising outcomes. However, we observe that Spikformer may exhibit excessive energy consumption, potentially attributable to redundant channels and blocks.

**Methods:**

To mitigate this issue, we propose a one-shot Spiking Transformer Architecture Search method, namely Auto-Spikformer. Auto-Spikformer extends the search space to include both transformer architecture and SNN inner parameters. We train and search the supernet based on weight entanglement, evolutionary search, and the proposed Discrete Spiking Parameters Search (DSPS) methods. Benefiting from these methods, the performance of subnets with weights inherited from the supernet without even retraining is comparable to the original Spikformer. Moreover, we propose a new fitness function aiming to find a Pareto optimal combination balancing energy consumption and accuracy.

**Results and discussion:**

Our experimental results demonstrate the effectiveness of Auto-Spikformer, which outperforms the original Spikformer and most CNN or ViT models with even fewer parameters and lower energy consumption.

## 1 Introduction

Spiking neural networks (SNNs) show promise for the next generation of artificial intelligence, owing to their biological inspiration and appealing features such as sparse activation and temporal dynamics. The performance of SNNs has improved by employing advanced architectures from ANNs, such as ResNet-like SNNs (Fang et al., 2021a; Hu et al., 2021a,b; Zheng et al., 2021), or Spiking Recurrent Neural Networks (Lotfi Rezaabad and Vishwanath, 2020). Transformer, originally developed for natural language processing (Vaswani et al., 2017), has proven successful in various computer vision applications, including image classification (Dosovitskiy et al., 2020; Yuan et al., 2021a), object detection (Carion et al., 2020; Zhu et al., 2020; Liu et al., 2021), and semantic segmentation (Wang et al., 2021; Yuan et al., 2021b). The self-attention mechanism, a crucial component of the Transformer model, selectively attends to relevant information and is analogous to an important feature of the human biological system (Caucheteux and King, 2022; Whittington et al., 2022). The integration of self-attention into SNNs for advanced deep learning has gained attention due to the biological properties of both mechanisms. Spikformer (Zhou et al., 2022), a recent SNN architecture, has demonstrated promising results on both static and neuromorphic datasets using its Spiking Self-Attention (SSA) and Spiking Patch Splitting (SPS) modules.

**Figure 1:**
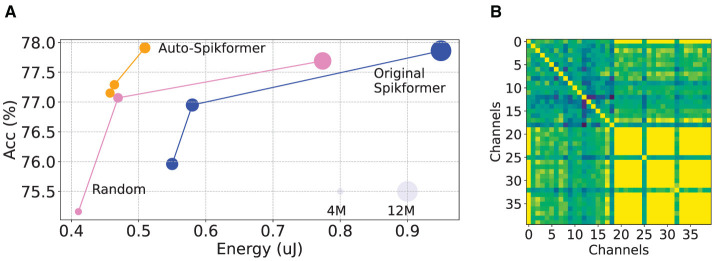
Analysis of redundancy of Spikformer **(A)** Relationship among energy consumption, number of parameters, and accuracy for various Spikformer candidates. Original Spikformer candidates are obtained from Zhou et al. (2022). We select 100 candidates from the Spikformer large search space $S_{T_{s}}$ using our proposed Auto-Spikformer method and random selection method, then plot their Pareto frontier onto the figure. Note that larger circles represent a higher number of parameters. Detailed results can be found in Section 5.3. **(B)** The Structural Similarity (SSIM) matrix between channels after embedding (also called SPS) in Spikformer. Both the X and Y axes represent channels. The color indicates the SSIM value: yellow denotes higher similarity, while green denotes lower similarity. The matrix reveals significant redundancy in channels, particularly in higher-order channels, after embedding.

While SNNs are known for their low energy consumption compared to ANNs, our observations revealed that the energy consumption of Spikformer can be significantly reduced as it contains potentially redundant channels and blocks. In Figure 1, we observed suboptimal architecture parameters in the original Spikformer, with redundancy channels, particularly in higher-order channels (See Section 3 for more details). These phenomenons motivates us to search to design a better Spikformer architectures. Nevertheless, designing and training such hybrid models remains a challenging task (Dosovitskiy et al., 2020; Touvron et al., 2021).

We address the Spikformer search problem by dividing it into two main parts: the Transformer part and the SNN neuron part. Transformer Architecture Search (TAS) (Chen B. et al., 2021; Chen M. et al., 2021; Su et al., 2022) has gained attention as an automated way to search for multiple configurations of Vision Transformer (ViT) architectures. The one-shot NAS scheme (Dong and Yang, 2019; Chen M. et al., 2021), leveraged in TAS, obtains reliable performance estimations on various ViT architectures. We choice weight entanglement supernet training strategy (Chen M. et al., 2021) as base search method to optimize the Transformer architecture. However, directly applying TAS may not be the most optimal solution for Spiking Transformers. The original TAS method does not consider the SNN search space and the energy consumption, which is vital in the field of SNN.

To optimize the internal parameters of SNN neurons, we propose a method that leverages the concept of natural selection and evolutionary algorithms. While previous studies have focused on improving SNN performance through network structure exploration, the significance of individual neuron parameters has also been identified (Che et al., 2022; Kim et al., 2022; Na et al., 2022). We draw inspiration from Darwin's theory of evolution, which suggests that organisms adapt to their environment through natural selection over time (Slowik and Kwasnicka, 2020; Jordan et al., 2021). Similarly, SNN neurons can evolve and optimize their internal parameters to enhance network performance. By treating traits such as the threshold, decay, and time-step parameters of a neuron as candidate solutions and the input stimuli as the environment, we can apply simulated evolution to find optimal parameter sets that improve accuracy and efficiency. This novel approach, referred to as Discrete Spiking Parameters Search (DSPS), utilizes an evolutionary algorithm to search for the internal parameters of SNN neurons. Our study is the first to apply the evolutionary algorithm to search for the internal parameters of SNN neurons.

Our method for optimizing Spikformer explores the optimal combination of key factors but doesn't ensure lower energy consumption. To address this, we introduce a joint fitness function, $F_{AEB}$, balancing energy consumption and accuracy. This allows us to achieve a Pareto optimal combination, striking a balance between these two objectives.

We summarize our contributions are as follows:

- We provide the first systematic and in-depth analysis of the channel redundancy in SNNs by analysing the performance curve and Structural Similarity (SSIM), which are crucial to the high energy efficiency.
- To the best of our knowledge, this study is the first to use NAS for spiking-based ViT, namely Auto-Spikformer. By employing Discrete Spiking Parameters Search (DSPS) and the weight entanglement supernet training method, Auto-Spikformer enhances the efficiency and accuracy of spiking-based ViT architectures.
- Auto-Spikformer integrates an accuracy and energy balanced fitness function $F_{AEB}$ to optimize the Spikformer search space by considering both energy consumption and accuracy simultaneously.

## 2 Related work

### 2.1 Spiking neural networks

Unlike traditional deep learning models that perform computations using floating-point values, SNNs leverage discrete spike sequences for information processing and transmission. Spiking neurons endow SNNs with temporal dynamics and biological properties. Common types include the leaky integrate-and-fire (LIF) neuron (Wu et al., 2018), PLIF (Fang et al., 2021b), etc. Two main approaches for obtaining deep SNNs are ANN-to-SNN conversion and direct training. In ANN-to-SNN conversion, a pre-trained ANN with high performance is transformed into an SNN by substituting the ReLU activation layers with spiking neurons (Cao et al., 2015; Hunsberger and Eliasmith, 2015; Rueckauer et al., 2017; Bu et al., 2021; Meng et al., 2022; Wang et al., 2022). However, this method requires large time-steps to approximate ReLU activation accurately, leading to high latency (Han et al., 2020). In direct training, SNNs are trained by backpropagation through time (BPTT) (Werbos, 1990). A challenge for direct training is the non-differentiability of the event-triggered mechanism in spiking neurons. To address this challenge, surrogate gradients are employed for backpropagation (Neftci et al., 2019; Lee et al., 2020; Xiao M. et al., 2021) adopts implicit differentiation on the equilibrium state to train SNNs.

### 2.2 Vision transformer

The Vision Transformer (ViT) facilitates the transformation from NLP to CV by partitioning visual information into patches and processing it accordingly. For image classification, a Transformer encoder comprises a patch splitting module, multiple Transformer encoder blocks, and a linear prediction head. Each Transformer encoder block includes a self-attention layer and a multi-perception layer. Self-attention is a fundamental component contributing to ViT's success. It captures global dependence and interest representation by weighing feature values of image patches via the dot product of the query and key, followed by the application of the softmax function (Katharopoulos et al., 2020; Qin et al., 2022). Researchers have made improvements to the visual transformer, including the Transformer architecture (Hassani et al., 2021; Xiao T. et al., 2021), more advanced self-attention mechanisms (Choromanski et al., 2020; Rao et al., 2021; Song, 2021; Yang et al., 2021), and pre-training techniques (He et al., 2022), among others. Spikformer (Zhou et al., 2022), a recent SNN-based Transformer, has demonstrated promising results on both static and neuromorphic datasets. Observations reveal potential redundancy in channels and blocks, motivating us to explore a more efficient SNN-based Transformer automatically.

### 2.3 One-shot NAS

Designing high-performance network architectures for specific tasks often requires expert experience and trial-and-error experiments. Neural architecture search (NAS) (Elsken et al., 2019) aims to automate this manual process and has recently achieved highly competitive performance in tasks such as image classification (Zoph and Le, 2016; Liu C. et al., 2018; Pham et al., 2018; Zoph et al., 2018; Real et al., 2019), object detection (Zoph et al., 2018; Chen Y. et al., 2019; Guo et al., 2020; Wang et al., 2020), and semantic segmentation (Liu et al., 2019; Nekrasov et al., 2019; Zhang et al., 2019; Lin et al., 2020), etc. However, searching over a discrete set of candidate architectures often results in a massive number of potential combinations, leading to explosive computational cost. The recently proposed differentiable architecture search (DARTS) method (Liu H. et al., 2018) and its variations (Chen X. et al., 2019; Xu et al., 2019; Chu et al., 2020) address this problem using a continuous relaxation of the search space, enabling learning a set of architecture coefficients by gradient descent. They have achieved competitive performances with the state-of-the-art using orders of magnitude fewer computation resources (Liu H. et al., 2018; Liu et al., 2019; Cheng et al., 2020). Recently, Na et al. (2022) studied pooling operations for downsampling in SNNs and applied NAS to reduce the overall number of spikes. Kim et al. (2022) applied NAS to improve SNN initialization and explore backward connections. However, both works only searched for different SNN cells or combinations of them within traditional CNNs. There is a lack of work on searching for SNN internal parameters and SNN-based transformer architectures.

## 3 Problem analysis

We conducted several experiments and metrics to analyze the redundancy in Spikformer. Our observations revealed three key phenomena: (1) the original Spikformer architecture parameters are not optimal; (2) there is redundancy in the channels after embedding; and (3) most of the redundant channels are found at higher-order channels.

**Exploration of the optimal combination of key factors in Spikformer**. As depicted in Figure 1A, we trained a supernet and randomly selected candidates to evaluate their performance, plotting their Pareto frontier. Surprisingly, some randomly selected candidates performed optimally in both energy and accuracy. Upon analyzing these high-performing candidates, we discovered that their blocks and channels were both fewer than those in the original Spikformer, indicating that the original architecture parameters are suboptimal.

**Analysis of redundancy in Spikformer**. Previous work has identified high sparsity and redundancy in spike features in spiking convolutional neural networks (Yao et al., 2023). Spiking Transformer also exhibits redundancy in both channels and the number of blocks. We further analyzed the Structural Similarity (SSIM) to measure the similarity between features at different scales. As shown in Figure 1B, we inferred the trained 8–384 Spikformer model and calculated SSIM between each channel's feature map after embedding. The feature map consists of 384 channels. We selected the top 20 channels with the highest and lowest SSIM scores, preserving their original order to construct a matrix. Our analysis revealed redundancy in the channels after embedding, with most of the redundant channels found at higher-order channels.

To address these issues, we propose a hybrid architecture search, using the Transformer Architecture Search (TAS) method to explore optimal combinations in Spikformer. In the TAS field, the weight entanglement method is used to train and select channels in order, as shown in Figure 4. Therefore, in this work, we use the weight entanglement method to optimize the transformer factors. However, this method cannot optimize discrete parameters like tau, threshold, and time-step, which are important in SNNs. Therefore, we propose a discrete method to optimize these spiking parameters, addressing the limitations in current optimization approaches.

## 4 Auto-Spikformer

We propose Auto-Spikformer, a one-shot Spiking Transformer Architecture Search method combining the search of Transformer and SNN neurons simultaneously. Auto-Spikformer consists of two stages: the supernet training stage and the evolutionary search stage. We first briefly introduce the spiking neuron, followed by an overview of Auto-Spikformer, the DSPS method, and the fitness function.

**Figure 2:**
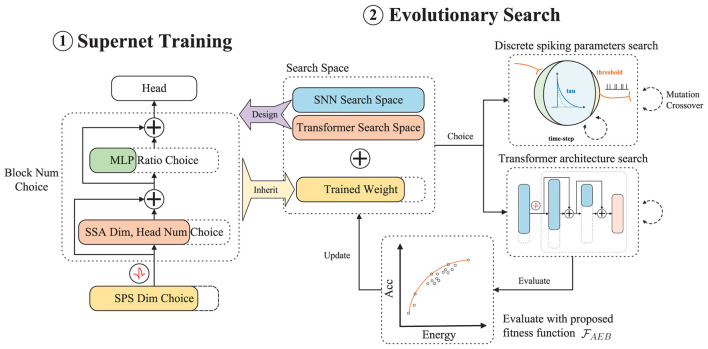
Auto-Spikformer including two stages: supernet training stage and evolutionary search stage. We first design a search space that includes both SNN inner parameters and transformer architecture. Within this search space, we train the supernet based on weight entanglement for the transformer space and an alternate choice method for the SNN space. After supernet training, we use evolutionary search to choose the best transformer architecture and optimal SNN inner parameters with weights inherited from the supernet. Note that we denote the process of designing the SNN search space, using the alternate choice method to train the SNN space supernet, and searching the SNN space with evolutionary search as Discrete Spiking Parameters Search (DSPS). We then evaluate the performance of the searched subnet with our proposed energy-accuracy balanced fitness function $F_{AEB}$. Spiking Self-Attention (SSA) is the attention block in Spikformer. And Spiking Patch Splitting (SPS) is the embedding module in Spikformer.

As shown in Figure 2, during the supernet training stage, we use Spikformer (Zhou et al., 2022) as our base model to construct the supernet. We then train the supernet using the weight entanglement method for the Transformer space and the alternate choice method for the SNN space. After supernet training, we employ evolutionary search to select the optimal transformer architecture and SNN inner parameters with weights inherited from the supernet (as discussed in Section 4.2). Note that the fitness function aims to find a Pareto optimal combination balancing energy consumption and accuracy, as shown in Section 4.4.

### 4.1 LIF

We adopt the iterative LIF neuron model (Wu et al., 2019) described by (Equation 1)


(1) $$\begin{array}{l} u^{t,n}=\left(1-\frac{1}{\tau }\right)u^{t-1,n}\left(1-y^{t-1,n}\right)+I^{t,n} \end{array}$$

where superscripts *n* and *t* denote the layer index and time-step, respectively. The decay τ is the membrane time constant, *u* is the membrane potential, *y* denotes the spike output, and *I* denotes the synaptic input with $I^{t,n}=\underset{j}{\sum }w_{j}y_{j}^{t,n-1}$, where *w* is the weight. The neuron fires a spike *y*^*t, n*^ = 1 when *u*^*t, n*^ exceeds a threshold *V*_*th*_; otherwise, *y*^*t, n*^ = 0. In this work, we set τ = 2 and *u*_*th*_ = 0.5.

### 4.2 Discrete spiking parameters search

**Motivation**. The performance of SNN neurons is influenced by both their interconnections and internal parameters. While previous research has primarily focused on enhancing SNN performance through modifications to the network's structure, the importance of optimizing the internal parameters within individual neurons cannot be overlooked. Darwin's theory of evolution posits that organisms adapt to their surroundings through natural selection, favoring traits that enhance survival and reproduction. This concept can be applied to the context of SNN, where individual neurons can undergo an evolutionary process. In this context, the internal parameters of a neuron, such as the threshold (*u*_*th*_), decay (τ), and time-step (*t*), can be seen as analogous to traits, while the input stimuli received by the neuron can be likened to the environment in which it operates. Previous work (Fontaine et al., 2014) suggests that the threshold can be viewed as an adaptation to membrane potentials at short timescales, influencing how signals received by a neuron are encoded into a spike. Decay τ has a similar effect to the threshold, but it only affects the decay of unfired neurons, influencing the firing of the next timestep. In contrast, the threshold affects the firing of all neurons at the current moment.

**Figure 3:**
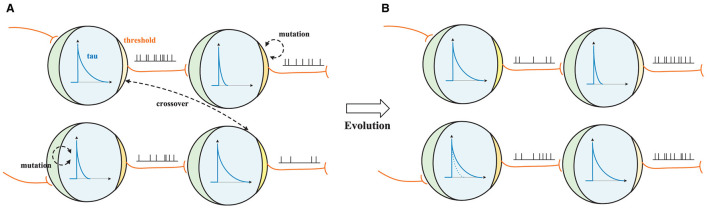
Discrete Spiking Parameters Search (DSPS) process. **(A, B)** show two candidate parameter sets before and after applying the mutation and crossover operators. The spike from the previous neuron is transmitted to the current neuron during the charging process. If the membrane potential is above the threshold (yellow area, where the darker color indicates a higher value), a spike is delivered; if the membrane potential is below the threshold, it decays exponentially with a time constant τ (blue curve). During the supernet training stage, these parameters are searched using the alternate choice method.

**Discrete spiking parameters search process**. As shown in Figure 3, the spike from the previous neuron is transmitted to the current neuron during the charging process. If the membrane potential is above the threshold, a spike is delivered; if the membrane potential is below the threshold, it decays at the rate of τ. The DSPS begins with a population of randomly generated parameter sets (candidates) like [*u*_*th*_ = 1.2, τ = 1.25, *t* = 4]. In each generation, the algorithm evaluates the fitness of candidates and selects the best ones as the parents for the next generation. The parents produce offspring by applying mutation and crossover operators with some probabilities. The mutation operator randomly modifies one parameter of a parameter set, while the crossover operator combines two parameters from different parents. As illustrated in Figure 3, for example, the decay τ of a candidate changes from 1.25 to 2.5 after mutation. The thresholds *u*_*th*_ of two candidates are swapped after crossover, affecting the firing rate of each candidate. The algorithm repeats this process for a fixed number of generations and returns the best architecture found. Through a process of simulated evolution, the threshold, decay, and time-step parameters of individual neurons can be adjusted to improve the performance of the network as a whole. According to our experiments, this approach can lead to the network becoming better adapted to the input stimuli it receives, resulting in increased accuracy and efficiency.

**Figure 4:**
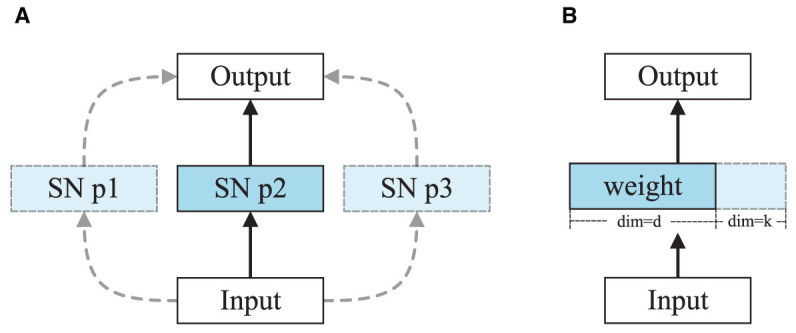
**(A)** Alternate choice method for training SNN search space. SN p1 denotes spiking neuron parameter setting 1, like [*u*_*th*_ = 1.2, τ = 1.25, *t* = 4]. Note that they are all discrete values and without trainable parameters. **(B)** Weight entanglement method for training transformer search space (Chen M. et al., 2021).

**Alternate choice and weight entanglement**. Figure 4 illustrates the differences between the weight entanglement method and the alternate choice method. The weight entanglement method allows different transformer blocks within a supernet to share weights for common parts in each layer. This strategy leads to faster convergence, lower memory cost, and better performance of subnets compared to classical weight-sharing methods. We apply this strategy to train the transformer block in Spikformer. However, the SNN search space is discrete and lacks trainable parameters, limiting the application of the weight entanglement method. It requires the search space to undergo continuous changes, or in other words, the search space should share some common parts; it cannot be entirely discrete. When we use weight entanglement, channels change from 380 to 384, with the previous 380 channels sharing the same weights, leaving only the last 4 channels different. However, for SNN inner parameters such as threshold, changing from 0.3 to 0.5, there are no common parts to share. Therefore, we employ the alternate choice method for training instead of weight entanglement.

### 4.3 Search space

We design a search space including both SNN inner parameters and transformer architecture as shown in Table 1.

**Table 1:** Auto-Spikformer search space.

	** $S_{T_{s}}$ **	** $S_{T_{l}}$ **
**(A) Transformer search space**		
Embed dim	(336,384,12)	(336,480,48)
MLP ratio	(3,4,0.2)	(3,5,0.2)
Head num	(6,12,6)	(6,12,6)
Depth	(2,4,1)	(2,6,1)
**(B) SNN search space**		
16.5-1,15.5242pt	$S_{S}$	
Threshold *u*_*th*_	(0.6,2,0.2)	
Decay τ	(1.25,10,0.25)	
Time-step *t*	(2,4,1)	

$S_{T_{s}}$ denotes the transformer smaller search space, $S_{T_{l}}$ denotes the transformer larger search space, and $S_{S}$ denotes the SNN search space. Each element in the table represents the lower limit, upper limit, and step size, such as depth (2,4,1) represents the depth range from 2 to 4, step size is 1.

**SNN search space**, denoted $S_{S}$, includes three variable factors: the threshold *u*_*th*_, decay τ, and time-step *t*. The structured definition of this search space is outlined in Table 1, and its visual interpretation is depicted in Figure 3.

**Transformer search space**, denoted $S_{T}$, is similar to the design of Autoformer (Chen M. et al., 2021), which includes four variable factors: embedding dimension, MLP ratio, head number, and depth. The structured definition of this search space is outlined in Table 1.

### 4.4 Accuracy and energy balanced fitness function ($F_{AEB}$)

The original fitness function only considers accuracy. We propose a new fitness function $F_{AEB}$ that balances accuracy and energy consumption. To estimate energy use, we first need to compute synaptic operations (SOPs). For a specific layer *l*, SOPs can be calculated as follows (Equation 2):


(2) $$\begin{array}{l} \text{SOPs}\left(l\right)=fr\times t\times \text{FLOPs}\left(l\right) \end{array}$$

Here, *fr* denotes the firing rate of the input spike train, and *t* represents the time-step. Floating Point Operations (FLOPs) refer to the number of multiply-and-accumulate (MAC) operations, while SOPs contain spike-based accumulate (AC) operations only. The theoretical energy consumption of Auto-Spikformer, assuming implementation on the 45nm CMOS technology (Rathi and Roy, 2021) with *E*_*MAC*_ = 4.6*pJ* and *E*_*AC*_ = 0.9*pJ*, is calculated as (Equation 3):


(3) $$\begin{array}{l} E=E_{MAC}\times {\text{FL}}_{\text{SNN Conv}}^{1}+E_{AC}\times (\sum_{n=2}^{N}{\text{SOP}}_{\text{SNN Conv}}^{n}\\ \;+\sum_{m=1}^{M}{\text{SOP}}_{\text{SNN FC}}^{m}+\sum_{l=1}^{L}{\text{SOP}}_{\text{SSA}}^{l}) \end{array}$$

Here, **E** denotes the model energy, ${\text{FL}}_{SNNConv}^{1}$ is the first layer to encode static RGB images into spike-form, and the SOPs of *n* SNN Conv layers, *m* SNN Fully Connected Layer (FC), and *l* SSA are added together and multiplied by *E*_*AC*_. For ANNs, the theoretical energy consumption of block *b* is calculated (Equation 4):


(4) $$\begin{array}{l} \text{Power}\left(b\right)=4.6pJ\times \text{FLOPs}\left(b\right) \end{array}$$

For SNNs, Power(*b*) is (Equation 5):


(5) $$\begin{array}{l} \text{Power}\left(b\right)=0.9pJ\times \text{SOPs}\left(b\right) \end{array}$$

The energy consumption of Spikformer is influenced by factors such as input image size, embedding dimension, number of blocks, firing rate *fr*, and time-step *t*. These factors can be adjusted by changing the transformer architecture and selecting suitable spike neuron parameters. For comparison, we normalized these factors using a minmax scaler and assigned different weights to both metrics. The accuracy and energy balanced fitness function $F_{AEB}$ is described as follows (Equation 6):


(6) $$\begin{array}{l} F_{AEB}=\alpha \times \text{E}+\left(1-\alpha \right)\times A \end{array}$$

Here, A denotes the top-1 accuracy, both metrics are scaled by a minmax scaler with the range (0,1), and α denotes the weight (set to 0.5 in our case).

## 5 Experiments

We provide comprehensive implementation details for the supernet training stage and the evolutionary search stage. Firstly, we evaluate the effectiveness of the proposed DSPS method by comparing it with random search and handcrafted design within the SNN search space. Then, we assess the effectiveness of the proposed $F_{AEB}$ fitness function within the Transformer and SNN mixed search space. Finally, we evaluate the performance of the searched model on the CIFAR dataset and neuromorphic datasets, comparing it with the original Spikformer and various CNN or ViT models.

### 5.1 Implementation details

**Supernet training stage**. We followed a similar training manner as Spikformer, with an extended epoch duration of 1000 to ensure improved convergence of the supernet.

**Evolutionary search stage**. For the transformer search space, we adopt a similar approach to Autoformer. In the SNN search space, our proposed DSPS begins with 50 randomly generated sets, each specifying the decay rate (τ), threshold (*u*_*th*_), and time-step (*t*). The fitness ($F_{AEB}$) is evaluated on test data, with the top 20 selected as parents. Offspring are generated through mutation and crossover, utilizing probabilities Pd (0.3) and Pm (0.4). This process iterates for 20 generations, culminating in the identification of the optimal architecture.

**Dataset**. We conducted our experiments on the CIFAR dataset and neuromorphic datasets. **CIFAR** consists of 50,000 training and 10,000 test images with a resolution of 32 × 32 pixels. **CIFAR10-DVS** is a neuromorphic dataset derived from the CIFAR10 static image dataset, comprising 9,000 training and 1,000 test images with a resolution of 128 × 128 pixels. **DVS128 Gesture** is a gesture recognition dataset consisting of 11 hand gesture categories with a resolution of 128 × 128 pixels.

### 5.2 Effectiveness of DSPS

**Figure 5:**
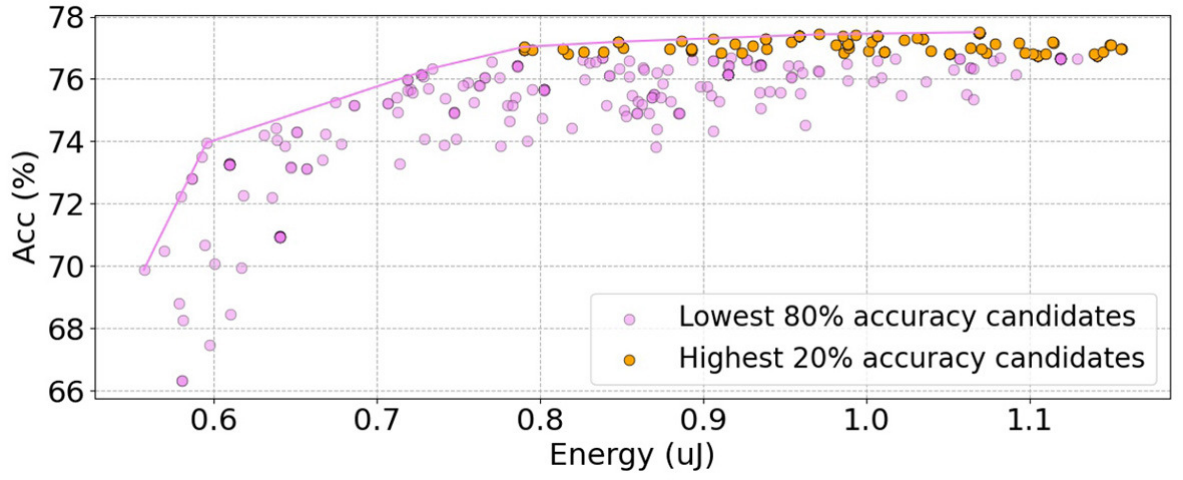
The energy and accuracy of all candidates in $S_{S}$ in CIFAR100. We use $F_{AEB}$ as the fitness function to select the top 300 candidates. The purple points represent the candidates with the lowest 80% accuracy, while the orange points represent the candidates with the highest 20% accuracy. The purple line represents the Pareto frontier, indicating the optimal trade-off between accuracy and energy consumption.

We train Auto-Spikformer within the SNN search space ($S_{S}$), where only the SNN parameter sets are modified while maintaining the original Spikformer structure depicted in Table 1. We select 300 candidates through the proposed DSPS and the $F_{AEB}$. We then plot energy and accuracy for each candidate and draw a Pareto frontier, as shown in Figure 5. Notably, by solely modifying the SNN inner parameter sets, a superior trade-off between energy consumption and accuracy can be achieved.

There is a moderate Kendall's tau rank correlation of 0.4 between the accuracy and the energy consumption. Some candidates exhibit lower energy consumption but higher accuracy, indicating that they are more optimal than others. The energy consumption within $S_{S}$ is mainly determined by the firing rate, as the architecture is fixed. We select the candidates located on the Pareto frontier, as well as a subset of candidates with the top 20% accuracy, and present them in Table 2.

**Table 2:** Subsets of the candidates in $S_{S}$.

**Candidates** **(threshold × 4, tau × 4, time-step)**	**Fr**	**Energy ** **(*μJ*)**	**Acc** **(%)**
**(A) Candidates on the Pareto frontier**			
(1.6, 0.6, 0.8, 2.0, 10, 10, 10, 2, 2)	0.20	0.52	72.12
(1.8, 0.8, 1.4, 1.2, 10, 10, 10, 2, 2)	0.20	0.52	72.18
(1.6, 0.6, 2.0, 1.8, 5, 10, 10, 1.5, 4)	0.24	0.63	75.58
(1.0, 1.0, 1.4, 1.0, 10, 10, 2, 3, 4)	0.25	0.66	76.44
(1.8, 1.6, 0.6, 0.8, 5, 10, 2, 3, 4)	0.26	0.68	76.77
(0.8, 1.2, 1.6, 2.0, 5, 10, 2, 1.5, 4)	0.27	0.72	77.20
(1.0, 2.0, 1.6, 2.0, 5, 2, 2, 3, 4)	0.30	**0.79**	**77.87**
(1.0, 2.0, 1.4, 1.6, 2, 2, 1.25, 5, 4)	0.37	0.99	77.95
(1.0, 1.0, 1.0, 1.0, 2, 2, 2, 2, 4)^*^	0.35	0.95	77.86
**(B) Candidates with the top 20% accuracy**			
(1.0, 2.0, 1.6, 2.0, 5, 2, 2, 3, 4)	0.30	**0.79**	**77.87**
(1.2, 1.8, 1.8, 1.6, 2, 10, 1.5, 5, 4)	0.33	**0.87**	**77.90**
(1.6, 1.2, 1.8, 2.0, 1.5, 5, 10, 2, 4)	0.33	**0.88**	**77.86**
(1.6, 0.8, 1.2, 1.8, 2, 10, 1.25, 3, 4)	0.34	0.91	77.74
(0.6, 1.4, 1.2, 0.6, 2, 3, 1.25, 5, 4)	0.35	0.94	77.78
(0.8, 0.8, 1.8, 1.8, 2, 2, 1.5, 2, 3)	0.36	**0.95**	**77.92**
(1.0, 1.4, 1.8, 0.6, 2, 2, 1.5, 3, 4)	0.36	0.96	77.77
(1.0, 2.0, 1.4, 1.6, 2, 2, 1.25, 5, 4)	0.37	0.99	77.95
(1.0, 1.0, 1.0, 1.0, 2, 2, 2, 2, 4)^*^	0.35	0.95	77.86

Each selected candidate includes 9 features: (threshold × 4, tau × 4, time-step). Using the first candidate as an example, (1.6, 0.6, 0.8, 2.0) means the thresholds of SNN for four blocks are (1.6, 0.6, 0.8, 2.0) separately. Fr denotes the firing rate. The last row corresponds to Spikformer 4-384, denoted as *, obtained by running the open-source code of Spikformer. The bold font represents that the energy consumption and accuracy are superior to Spikformer 4-384.

We observe that our fitness function and search algorithm favor a time-step of 4, which is the maximum value in $S_{S}$. Furthermore, we aim to understand why different levels of energy consumption can result in similar accuracy. We notice that the network weights of these candidates are identical. Among them, the minimum energy consumption recorded is 0.79, while the maximum energy consumption is 0.99, resulting in a 25% difference. Remarkably, despite this significant divergence in energy consumption, the corresponding accuracies achieved are nearly equivalent.

As shown in Table 2, for a similar threshold value, the firing rate decreases as the decay parameter increases. The evolutionary search tends to adjust the tau parameter rather than the threshold to control the firing rate. The decay parameter in SNN has a profound effect on the firing rate by facilitating a memory effect for the previous membrane potential. Additionally, the decay and threshold parameters also affect the distribution of feature maps across the layers. Thus, by adjusting the tau and threshold values of each neuron, we can alter the firing rate and accuracy substantially. This shows that the proposed DSPS is a promising approach. By designing an appropriate search space and selecting a suitable fitness function, we are able to effectively decrease the overall firing rate while preserving the network's performance.

### 5.3 Effectiveness of $F_{AEB}$

To demonstrate the superiority of $F_{AEB}$, we conduct extensive experiments and illustrate the trade-off between energy and accuracy. We apply evolutionary search with $F_{AEB}$ as the fitness function to generate 1000 samples in both $S_{T_{s}}$ and $S_{T_{l}}$. We then select the top 100 candidates based on their scores. For comparison, we also randomly sample 100 candidates from the search space. Additionally, we include the Spikformer architecture in the energy-accuracy plot.

**Figure 6:**
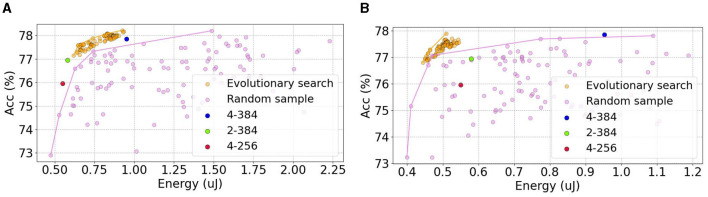
The energy and accuracy of all searched candidates in $S_{T_{s}}$ **(A)** and $S_{T_{l}}$ **(B)** in CIFAR100. We use $F_{AEB}$ as the fitness function (purple points) and randomly (orange points) select the 100 candidates, respectively. The other color points represent the different architectures of Spikformer, derived from the original paper (Zhou et al., 2022).

As shown in Figure 6, the Pareto front of $F_{AEB}$ dominates the random sample approach. The Kendall's tau rank correlation coefficients of evolutionary search and random sample are 0.63 and 0.08 in $S_{T_{s}}$ and 0.60 and 0.24 in $S_{T_{l}}$, respectively. The candidates on the Pareto front are listed in Table 3.

**Table 3:** Subsets of the candidates in $S_{T}$.

	**Candidates (depth (d), MLP ratio × d, head num × d, threshold × d, tau × d, time-step, embed dim)**	**Energy (*μJ*)**	**Acc (%)**
**(A) Candidates in** $S_{T_{s}}$**. We denote** ^1, 2^ **as Auto-Spikformer** $S_{T_{s}1,2}$.			
Pareto frontier	(2, 3.2, 3.0, 12, 6, 1.2, 1.0, 5, 5, 4, 348)	0.448	76.89
	(2, 3.4, 3.2, 12, 6, 1.0, 1.0, 5, 5, 4, 348)	0.453	77.04
	(2, 3.8, 3.2, 12, 6, 0.6, 1.8, 5, 5, 4, 348)	0.458	77.15
	(2, 3.8, 3.8, 12, 6, 1.0, 1.8, 5, 5, 4, 348)	0.464	77.29
	(2, 3.6, 3.6, 6, 12, 1.8, 2.0, 5, 2, 4, 348)	**0.509**	**77.91**
Top accuracy	(2, 3.8, 3.6, 12, 12, 1.4, 2.0, 5, 2, 4, 348)^1^	0.505	77.71
	(2, 3.6, 3.6, 6, 12, 1.8, 2.0, 5, 2, 4, 348)^2^	**0.509**	**77.91**
	(2, 3.6, 3.6, 6, 12, 0.6, 2.0, 5, 2, 4, 348)	**0.510**	**77.91**
	(2, 3.4, 3.8, 6, 6, 1.0, 1.8, 5, 2, 4, 360)	**0.535**	**77.89**
	(2, 3.6, 3.6, 6, 6, 0.6, 2.0, 5, 2, 4, 360)	**0.536**	**77.90**
Spikformer 4-384	(4, 4, 4, 4, 4, 12, 12, 12, 12, 1.0, 1.0, 1.0, 1.0, 2, 2, 2, 2, 4, 384)	0.95	77.86
**(B) Candidates in** $S_{T_{l}}$**. We denote** ^1, 2, 3^ **as Auto-Spikformer** $S_{T_{l}1,2,3}$.			
(2, 4.8, 3.2, 6, 6, 1.4, 1.6, 5, 5, 4, 384)		0.619	77.15
(2, 3.8, 3.2, 6, 12, 0.8, 1.2, 5, 5, 4, 432)		0.647	77.59
(2, 3.8, 4.2, 12, 6, 1.4, 1.4, 3, 5, 4, 432)		**0.737**	**77.88**
(2, 3.8, 4.2, 12, 12, 0.8, 1.2, 3, 1.5, 4, 432)		**0.829**	**78.08**
(3, 3.2, 3.6, 3.2, 6, 12, 6, 1.4, 1.8, 0.8, 3, 5, 5, 4, 480)		**0.925**	**78.22**
(2, 3.8, 3.2, 6, 12, 0.8, 1.2, 3, 1.5, 4, 432)^1^		**0.826**	**78.01**
(2, 3.8, 4.2, 12, 12, 0.6, 0.8, 3, 1.5, 4, 432)		**0.829**	**78.08**
(2, 4.2, 4.2, 12, 12, 0.8, 1.2, 3, 1.5, 4, 480)^2^		**0.889**	**78.05**
(3, 3.2, 3.6, 3.2, 6, 12, 6, 1.4, 1.8, 0.8, 3, 5, 5, 4, 480)^3^		**0.925**	**78.22**
(3, 3.2, 3.6, 3.0, 6, 12, 12, 1.4, 2.0, 2.0, 3, 5, 3, 4, 480)		**0.934**	**78.17**
(4, 4, 4, 4, 4, 12, 12, 12, 12, 1.0, 1.0, 1.0, 1.0, 2, 2, 2, 2, 4, 384)		0.95	77.86

Each selected candidate includes *d*×4+3 features: (depth (d), MLP ratio × d, head num × d, threshold × d, tau × d, time-step, embed dim). Using the first candidate as an example, (2) denotes there are two only blocks. (3.2, 3.0) means MLP ratio for two blocks is (1.6, 0.6, 0.8, 2.0) separately. Blue color represents the SNN search space while orange color represents the transformer search space. Bold value means the result is more optimal.

We observe numerous candidates that achieve a favorable balance between accuracy and energy consumption. In $S_{T_{s}}$, some candidates on the frontier even surpass the original 4–384 Spikformer architecture in accuracy with only 2 blocks and 348 channels, meaning half of the energy consumption. $S_{T_{l}}$ is used to further explore higher accuracy architecture, as shown in Table 3. The highest accuracy is 78.22 with lower energy consumption of 0.925 μ*J*. Furthermore, several candidates exhibited 10% to 25% less energy while achieving higher accuracy compared to the 4–384 Spikformer architecture.

### 5.4 Results on CIFAR

We select the Auto-Spikformer architecture searched in $S_{T_{s}}$ and $S_{T_{l}}$ in Section 5.3 and compare it with the original Spikformer and other methods. The performances are reported in Table 4. Auto-Spikformer is the first transformer model designed through automated methods. AutoST is another research work conducted during the same period, also focuses on spiking transformers but uses a training-free method to obtain suitable architecture candidates, which are then retrained from scratch. In contrast, our approach involves training a supernet and extracting candidates without the need for retraining. In terms of performance, AutoST's optimal architecture increases the number of parameters by nearly 3.5 times compared to our optimal model, achieving only a 1.5% accuracy improvement. Additionally, their minimum model's performance is lower than ours, with a 1.42% accuracy difference while using nearly the same number of parameters. Auto-Spikformer $S_{T_{s}2}$ and $S_{T_{l}1,2,3}$ outperform the state-of-the-art methods, including CNN or Transformer models that are manually or automatically designed, in both accuracy and energy consumption. The ANN-Transformer model is only 0.34% and 2.8% better than $S_{T_{l}1,2,3}$ in CIFAR10/100, respectively, demonstrating that the Auto-Spikformer method is comparable to the ANN version.

**Table 4:** Performance comparison of Auto-Spikformer with existing methods on CIFAR10/100.

**Methods**	**Architecture**	**Param (M)** **/ Energy (*μJ*)**	**Time** **step**	**CIFAR10** **Acc**	**CIFAR100** **Acc**	**Model** **type**	**Design** **type**
Hybrid training (Rathi et al., 2020)	VGG-11	9.27 / -	125	92.22	67.87	CNN	Manual
Diet-SNN (Rathi and Roy, 2020)	ResNet-20	0.27/ -	10**/**5	92.54	64.07	CNN	Manual
STBP (Wu et al., 2018)	CIFARNet	17.54 / -	12	89.83	-	CNN	Manual
STBP NeuNorm (Wu et al., 2019)	CIFARNet	17.54 / -	12	90.53	-	CNN	Manual
TSSL-BP (Zhang and Li, 2020)	CIFARNet	17.54 / -	5	91.41	-	CNN	Manual
STBP-tdBN (Zheng et al., 2021)	ResNet-19	12.63 / -	4	92.92	70.86	CNN	Manual
TET (Deng et al., 2022)	ResNet-19	12.63 / -	4	94.44	74.47	CNN	Manual
AutoSNN (Na et al., 2022)	AutoSNN (C=128)	21 / -	8	93.15	69.16	CNN	Auto
SNASNet (Kim et al., 2022)	SNASNet-Bw	- / -	8	94.12	73.04	CNN	Auto
SpikeDHS^*D*^ (Che et al., 2022)	SpikeDHS-CLA (n3s1)	14 / -	6	95.36	76.25	CNN	Auto
ANN	ResNet-19*	12.63	1	94.97	75.35	CNN	Manual
	Transformer-4-384	9.32 / 3.97	1	**96.73**	**81.02**	Transformer	Manual
Spikformer	Spikformer-4-256	4.15 / 0.553	4	93.94	75.96	Transformer	Manual
	Spikformer-2-384	5.76 / 0.582	4	94.80	76.95	Transformer	Manual
	Spikformer-4-384	9.32 / 0.952	4	95.19	77.86	Transformer	Manual
AutoST	AutoST Tiny	4.20 / -	4	95.14	76.29	Transformer	Auto
	AutoST base	29.64 / -	4	96.21	79.69	Transformer	Auto
**Auto-Spikformer**	Auto-Spikformer $S_{T_{s}1}$	4.69 / **0.505**	4	**95.29**	**77.71**	Transformer	Auto
	Auto-Spikformer $S_{T_{s}2}$	4.64 / **0.509**	4	**95.23**	**77.91**	Transformer	Auto
	Auto-Spikformer $S_{T_{l}1}$	**7.09** / **0.826**	4	**96.19**	**78.01**	Transformer	Auto
	Auto-Spikformer $S_{T_{l}2}$	**9.20** / **0.889**	4	**96.38**	**77.05**	Transformer	Auto
	Auto-Spikformer $S_{T_{l}3}$	**8.46** / **0.925**	4	**96.39**	**78.22**	Transformer	Auto

Auto-Spikformer architectures $S_{T_{s}1,2}$ and $S_{T_{l}1,2,3}$ are selected from the candidate architectures listed in Table 3. Auto-Spikformer is the first transformer model designed through automated methods, demonstrating enhanced performance in both tasks. The symbol “*” denotes results obtained from self-implemented experiments by Deng et al. (2022). Bold value means the result is more optimal.

### 5.5 Results on neuromorphic datasets

As the dimensions and depth of neuromorphic datasets differ from the CIFAR dataset, we design a new search space for neuromorphic datasets. Following the same supernet training approach and evolutionary search manner as the CIFAR dataset, we report the results in Table 5.

**Table 5:** Comparison of the performance with state-of-the-art (SOTA) methods on two neuromorphic datasets.

**Method**	**Spikes**	**CIFAR10-DVS**		**DVS128**	
		***T* Step**	**Acc**	***T* Step**	**Acc**
LIAF-Net (Wu et al., 2021)	✗	10	70.4	60	97.6
TA-SNN (Yao et al., 2021)	✗	10	72.0	60	98.6
Rollout (Kugele et al., 2020)	✓	48	66.8	240	97.2
DECOLLE (Kaiser et al., 2020)	✓	-	-	500	95.5
tdBN (Zheng et al., 2021)	✓	10	67.8	40	96.9
PLIF (Fang et al., 2021b)	✓	20	74.8	20	97.6
SEW-ResNet (Fang et al., 2021a)	✓	16	74.4	16	97.9
Dspike (Li et al., 2021)	✓	10	75.4^*^	-	-
SALT (Kim and Panda, 2021)	✓	20	67.1	-	-
DSR (Meng et al., 2022)	✓	10	77.3^*^	-	-
Spikformer (Zhou et al., 2022)	✓	10	78.9^*^	10	96.9
	✓	16	80.9^*^	16	98.3
**Auto-Spikformer**	✓	16	**81.2** ^*^	16	**98.6**

Bold font indicates the best; ^*^ denotes with Data Augmentation.

It can be observed that our model achieves impressive performance on both datasets while utilizing a smaller model size (Spikformer is 2.59M, our optimal choice 2.48M) and less energy. Specifically, on the DVS128 Gesture dataset, we achieve an accuracy of 98.6% using 16 time steps. Furthermore, our results are competitive with the TA-SNN model (98.6%, 60 time steps) (Yao et al., 2021), which employs floating-point spikes in the forward propagation process. Also, on the CIFAR10-DVS dataset, our Auto-Spikformer model outperforms the state-of-the-art methods in terms of accuracy. Compared to the original Spikformer, the Auto-Spikformer achieves a significant improvement in accuracy with even less energy consumption.

## 6 Conclusion

In this work, we are the first to propose a one-shot spiking transformer architecture search method for spiking-based vision transformers, named Auto-Spikformer. Auto-Spikformer optimizes both energy consumption and accuracy by incorporating critical parameters of SNN and transformers into the search space. We introduce two novel methods: Discrete Spiking Parameters Search (DSPS), which optimizes SNN parameters, and the Accuracy and Energy Balanced Fitness Function $F_{AEB}$, designed to balance energy consumption and accuracy objectives. Extensive experiments demonstrate that the proposed algorithm significantly enhances the performance of Spikformer and uncovers numerous promising architectures. As part of our future work, we plan to extend our experiments to larger benchmark datasets.

## Data availability statement

The original contributions presented in the study are included in the article/supplementary material, further inquiries can be directed to the corresponding authors.

## Author contributions

KC: Conceptualization, Data curation, Formal analysis, Funding acquisition, Investigation, Methodology, Project administration, Resources, Software, Supervision, Validation, Visualization, Writing – original draft, Writing – review & editing. ZZ: Writing – original draft, Writing – review & editing. JN: Writing – review & editing. ZM: Resources, Writing – review & editing. WF: Methodology, Writing – review & editing. YC: Methodology, Writing – review & editing. SS: Validation, Writing – review & editing. LY: Funding acquisition, Investigation, Methodology, Writing – review & editing. YT: Funding acquisition, Writing – review & editing.
